# Leisure Physical Activity and the Risk of Fracture in Men

**DOI:** 10.1371/journal.pmed.0040199

**Published:** 2007-06-19

**Authors:** Karl Michaëlsson, Helena Olofsson, Karin Jensevik, Sune Larsson, Hans Mallmin, Lars Berglund, Bengt Vessby, Håkan Melhus

**Affiliations:** 1 Section of Orthopaedics, Department of Surgical Sciences, University Hospital, Uppsala, Sweden; 2 Uppsala Clinical Research Center, University Hospital, Uppsala, Sweden; 3 Section of Geriatrics, Department of Public Health, University Hospital, Uppsala, Sweden; 4 Section of Clinical Pharmacology, Department of Medical Sciences, University Hospital, Uppsala, Sweden; University of Edinburgh, United Kingdom

## Abstract

**Background:**

Data from previous studies are inconsistent, and it is therefore uncertain whether, to what extent, and at what level leisure physical activity influences the risk of osteoporotic fractures in men.

**Methods and Findings:**

A cohort of 2,205 men, 49–51 y of age, was enrolled in a longitudinal, population-based study. Leisure physical activity and other lifestyle habits were established at baseline and at ages 60, 70, 77, and 82 y. During 35 y of follow-up, 482 men had at least one fracture. Cox's proportional hazards regression was used to determine hazard ratios (HRs) of fracture associated with time-dependent physical activity habits and covariates. Men with a sedentary lifestyle (HR 2.56, 95% confidence interval 1.55–4.24) or men who walked or bicycled only for pleasure (HR 1.61, 95% confidence interval 1.10–2.36) had an increased adjusted risk of hip fracture compared with men who participated in regular sports activities for at least 3 h/wk. At the end of follow-up, 8.4% of the men with a high physical activity, 13.3% of the men with a medium physical activity, and 20.5% of the men with a low physical activity had suffered a hip fracture. According to the estimation of population-attributable risk, one third of all hip fractures could be prevented by participation in regular sports activities. High activity also conferred a reduced overall fracture risk.

**Conclusions:**

Our data indicate that regular sports activities can reduce the risk of fractures in older men.

## Abstract 2

From a large cohort study with 35 years of follow-up, Michaelsson and colleagues conclude that regular sport activities can reduce the risk of fractures in older men.

## Editors' Summary

**Background.:**

One of the hazards of old age is that the bones become less dense—and therefore weaker—so when an elderly person falls, the result is often a broken bone. As many as half of all women and a quarter of men older than 50 y will break a bone because of this, and the consequences can be serious, particularly if the hip is broken. The thinning of bones, which is known as osteoporosis, does affect all people as they age, but the degree to which it occurs varies greatly between individuals. A priority area for medical research is finding ways in which osteoporosis can be reduced, with the aim of improving the lives of older people and reducing their risk of “osteoporotic fractures.” It is known that genetic and environmental factors can both play a part in how rapidly osteoporosis develops, but it is generally agreed that personal lifestyle factors are also important. Osteoporosis develops over many years; in most people bone density starts to decline after the age of about 30 y. Preventive action should therefore begin early.

Most research so far has focused on women, who are more at risk as the thinning of their bones increases after the menopause. (Indeed osteoporosis has sometimes been wrongly described as a “woman's disease.”) It is now accepted that women who are more physically active reduce the rate of decline in their bone density and, as a result, are less likely to break bones when they are elderly. There has been little research in men and the results have not been consistent.

**Why Was This Study Done?:**

In order to provide better evidence as to whether men who do more physical activity have fewer osteoporotic fractures than those with lower activity levels, the researchers wanted to complete a study that was larger and was conducted over a longer period of time than previous research.

**What Did the Researchers Do and Find?:**

Between 1970 and 1973, the researchers invited all those men living in Uppsala, Sweden, who were aged between 49 and 51 y to participate in a health survey. Most of them (2,205) agreed to do so. When the study began, they were asked questions about the amount of physical activity they took outside working hours. They were asked the same questions again when they were aged 60, 70, 77, and 82 y. A record was also kept of the number of fractures the men had suffered during the 35-y study period. (Although some of the men died before the end of the study, about half were still alive at the end.) On the basis of the answers to the questions on physical activity at the start of the study, the researchers divided the men into three categories: those whose lifestyle was considered to be “sedentary,” those whose leisure activities included some walking and cycling, and those who participated in sports for at least 3 h a week. These were referred to as the low, medium, and high activity groups. Over the 35-y period, 428 men had at least one fracture and 134 broke a hip, but there were big differences between the groups—20% of the low-activity men had fractures compared with 13% of those with medium activity and only 8% of those in the high-activity group. In particular, the chance of having a hip fracture was reduced by increased activity.

**What Do These Findings Mean?:**

Taking exercise reduces the risk of an osteoporotic fracture. Participating in sports seems to be particularly effective; the researchers calculate that one-third of fractures could be prevented if men could be persuaded to take part in sports regularly. The researchers do note that the very best evidence always comes from studies where people are assigned at random to receive a particular “treatment” (in this case, it would be exercise) and are compared with others who did not receive the treatment. This is known as a “randomized controlled trial.” Such a trial would be difficult, if not impossible, to organize on this topic, and the approach adopted by the researchers, which is known as a “cohort study,” does provide very strong evidence. There are many other benefits from increased exercise (for example, in reducing the risk of heart attacks and strokes), and most governments are now promoting sports and other active leisure pursuits. This study adds further weight to support such policies.

**Additional Information.:**

Please access these Web sites via the online version of this summary at http://dx.doi.org/10.1371/journal.pmed.0040199.g002.

- There are many free sources of information about osteoporosis on the Web, and many organizations exist to support people with the condition. For example, the National Osteoporosis Society (UK) has useful information about the condition
- In the USA, there is the Nationtal Osteoporosis Foundation (USA)
- The equivalent organization in Australia is Osteoporosis Australia
- The UK National Health Service's NHS Direct Health Encyclopedia has an entry on osteoporosis
- MedlinePlus is an excellent source of information

## Introduction

Osteoporotic fractures, particularly hip fractures, constitute a large and growing problem worldwide, in both women and men, with a profound impact on quality of life [1] and mortality [2]. The fracture risk is influenced both by the genetic constitution and by environmental factors, with lifestyle becoming more important with increasing age [3].

Physical activity, one conceivable and modifiable risk factor, can prevent fractures by improving muscle mass and balance, and by increasing skeletal strength, and thus reducing the risk of injurious falls [4,5]. However, the clinical relevance regarding exercise for maintaining or improving bone mineral density in adult men cannot be determined from existing studies [6,7].

The investigation of the effects of physical activity on the most important outcome—fracture risk—should ideally be evaluated in a randomized study, but this design is unlikely to ever be well performed owing to methodological issues, e.g., study size, compliance, drop-outs, blinding and long-term follow-up. Therefore, it is not surprising that there are no randomized trials in this area.

Although moderate levels of leisure physical activity, such as walking, are associated with a substantially lower risk of hip fracture in postmenopausal women [8], data from prospective observational fracture studies in men are inconsistent. Whereas some studies in men report significant reductions in risk with a high physical activity [9–12], others do not [13–17]. Lack of validation and the absence of regular assessment of physical activity during follow-up may be factors that explain these contradictory results. The analyses in the positive reports have involved few osteoporotic fractures, and no consistent dose-response pattern has been detected. In addition, only a few studies have taken possible confounding by poor health into account, and in none of the studies has it been considered that changes in physical activity and other lifestyle habits might have occurred during follow-up. Thus, it is uncertain whether, to what extent, and at what level physical activity influences the risk of osteoporotic fractures in men.

We therefore investigated the impact of physical activity on the risk of fracture in a population-based cohort of men followed over a 35-y period.

## Methods

### Participants

From 1970 to 1973, all 2,841 men born between 1920 and 1924 and living in the municipality of Uppsala, Sweden, were invited to participate in a health survey, the Uppsala Longitudinal Study of Adult Men (ULSAM) [18]. A total of 2,322 men (82% of those invited), aged between 49 and 51 y, agreed to participate. Information regarding recreational physical activity was obtained by a reliability-tested questionnaire [19], but only 2,205 men (95%) responded to these questions, and it is these men who form the study base for the present investigation. At 60 y of age, 1,860 men took part in a second evaluation, at 70 y 1,221 men took part in a third evaluation, at 77 y 839 men participated and, at the final evaluation, at age 82 y, there were 530 participants. At the end of the follow-up period (December 31, 2006), 1,329 out of the 2,205 men (60%) had died. Thus excluding those who had died, at the beginning of each reinvestigation, the proportion of eligible participants who finally took part at the follow-up visits was 90%, 77%, 68% and, at the final investigation, 56%. The study was approved by the Ethics Committee at the Faculty of Medicine at Uppsala University, Sweden. Written informed consent was obtained from all participants.

### Physical Activity

Identical questions were asked at each of the five investigations: (1) Do you spend most of your time reading, watching TV, going to the cinema, or engaging in other, mostly sedentary, activities? (2) Do you often go walking or cycling for pleasure? (3) Do you engage in any active recreational sports or heavy gardening for at least 3 h every week? (4) Do you regularly engage in hard physical training or competitive sport? The answers are not mutually exclusive, but we considered the highest positive physical activity response level as prevailing. The highest two categories, questions 3 and 4, were considered together, since only 5% of the participants reported hard physical training. Therefore, based on the responses to the questions, three physical activity categories were constructed: low, medium, and high.

In conjunction with the third investigation, physical fitness was estimated by a symptom-limited exercise test on an ergometer cycle in 100 men [20]. The maximum exercise capacity was found to be associated with the reported physical activity level: for each higher physical activity category, the maximal workload increased on average by 12 W (*p* = 0.004), maximal oxygen uptake (VO_2_max) increased by 0.4 l/min (*p* = 0.03) [21], and VO_2_max/kg of bodyweight increased by 5 ml/min (*p* = 0.03). In addition, muscle biopsy specimens from the right vastus lateralis muscle were taken in a random sample of 475 men at the third investigation [22]. Compared with sedentary men, the men who reported moderate physical activity had 14% more capillaries per muscle fiber (*p* = 0.04), and those in the high activity group had 19% more capillaries per muscle fiber (*p* = 0.004), with an overall *p* for trend of <0.001.

Information on occupational physical activity was obtained through record linkage with national censuses from 1970 and 1980. Using job titles, the occupational physical demands were classified as very high or high, moderate, light, or sedentary [23].

### Identification of Fractures

We matched the study cohort to the national Hospital Discharge Register to identify cases of fractures treated on an inpatient basis [18]. All orthopedic records, radiographic records, and outpatient registers at the local hospitals were reviewed to identify fractures treated in outpatient departments.

### Statistical Analysis

The hip and overall fracture risk associated with the three leisure physical activity categories was analyzed. For these associations, we used time-dependent Cox proportional hazards regression to estimate hazard ratios (HRs), and 95% confidence intervals (CIs), for the physical activity levels. Thus, the values of physical activity, as well as covariate information, depended on follow-up time, i.e., the values were updated by data from each revisit. We performed all analyses by using the SAS PHREG procedure with delayed entry in the SAS software (version 9.1, SAS Institute, http://www.sas.com). The men contributed person-time from the date of the first investigation, until the date of a first fracture, death, or at the end of the follow-up period (December 31, 2006), whichever came first. Dates of deaths were based on data from the continuously updated Swedish National Population Register. Cases of fractures due to suspected high-impact trauma were retained in the analysis since there are indications of comparable increases in the risks of low- and high-impact trauma fractures in association with decreasing bone density in the elderly [24], but exclusion of these cases did not alter our results. We censored six cases of fracture caused by metastatic cancer.

We furthermore analyzed whether hip fracture risk was associated with changes in physical activity during the study. Changes in physical activity from age 50 to 60 y were considered in four categories: unchanged low and moderate activity, unchanged high activity, change from high to low-moderate, and change from low-moderate to high. Study entry for this particular analysis was age 60 y.

In an attempt to validate the robustness of the Cox's regression model, we used marginal structural modeling as described by Robins [25]. The relative risk estimates achieved with this latter approach were similar to the HRs computed with time-dependent Cox's regression analysis. Only the latter results are presented.

We considered a crude and a multivariable model including several time-dependent variables: weight and height (both continuous), smoking (never, former, current), perceived health (not good, good), physical activity at work in 1970 or 1980 (sedentary, light, medium, high, unknown, unemployed), and diabetes mellitus (yes/no), as well as musculoskeletal disorders, including rheumatoid arthritis and other inflammatory joint diseases (yes/no). Exposure in study participants who did not respond to this question at the follow-up visits was classified as being identical to that at the previous visit. In addition, we included in the model alcohol use at age 60 and at age 77, estimated by the Michigan Alcoholism Screening Test [26] (abstainer, normal, or suspected alcohol dependence).

Further adjustment, after additional matching to the inpatient register, was made for diseases which could theoretically influence physical performance and fracture risk: malignancies; endocrine disorders including hypogonadism; anemia and other blood diseases; any cardiovascular disease, psychiatric disorders such as depression, dementia, schizophrenia, alcohol and drug abuse; and infectious, neurological, respiratory, and gastrointestinal diseases (all yes/no). In the model, we also had the possibility to control for marital status (single, divorced/widowed, married/cohabitant), education (low, high, unclassified), age at study entry (continuous), duration of smoking (continuous), and self-reported disabilities such as aching joints (yes/no), regular use of pain killers (yes/no), chest pain (yes/no), or dyspnae (yes/no), back pain (yes/no), living under stress (yes/no), bad appetite (yes/no), and a fracture of the hip, spine, or lower forearm after the age of 40 y of the mother or father to the participants. We also included, as continuous variables, the daily intake of calories, vitamin D, calcium and alcohol, all calculated using a 7-d dietary record in conjunction with the third evaluation. All these additional adjustments influenced our fracture risk only marginally, and they were thus not retained in the final model.

The population-attributable risk of fracture among those with a specific physical activity level was calculated as

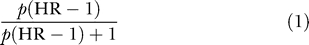
where *p* is the prevalence of the investigated physical activity level among the fracture cases [27].

## Results

The baseline characteristics of the men are presented by physical activity level in Table 1. Half of the men reported a high physical activity level. Current smoking was most frequent among sedentary men, but only minor differences in baseline anthropometric measures were observed between the physical activity categories.

**Table 1:**
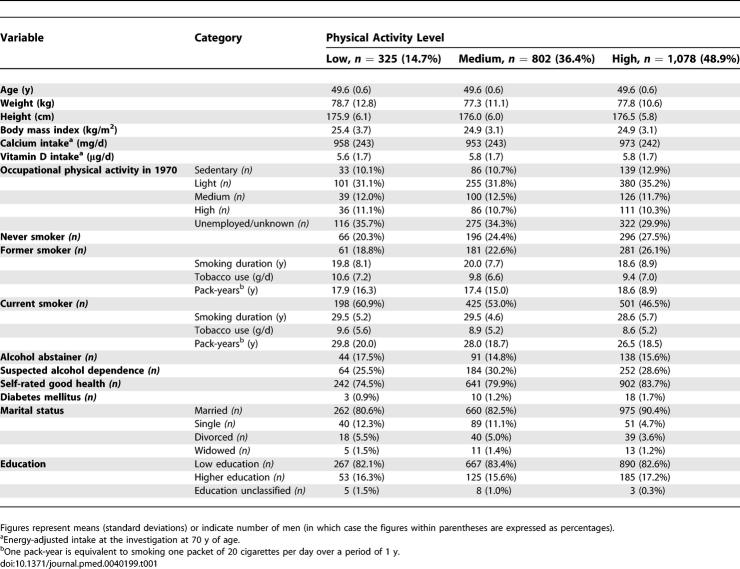
Characteristics at Baseline

**Figure 1:**
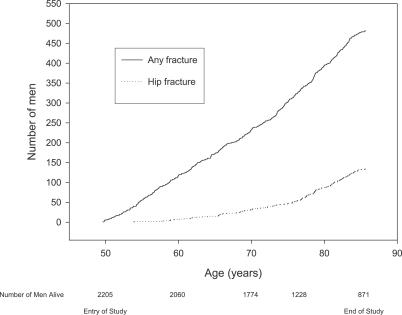
Cumulative Number of Hip Fractures and Fractures of any Type after Study Entry at Age 50 y and during Follow-up

During follow-up, we identified 482 men (22%) with any type of fracture and 134 men with hip fractures (6%) after study entry. Cumulative plots of when these two fracture categories occurred during follow-up is displayed in Figure 1. Although more than half of the men had died by the end of the study, we found a continuous increase with time in the absolute number of fractures.

**Figure 2:**
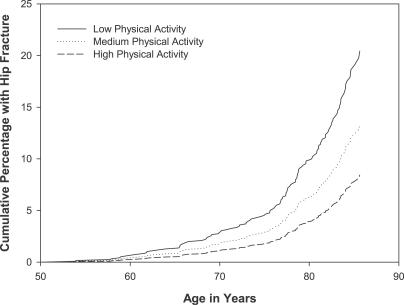
Survival Curves of Hip Fractures after Study Entry at 50 y of Age for the Time-Dependent Low, Medium, and High Physical Categories Survival curves are adjusted by changes in physical activity during follow-up.

**Figure 3:**
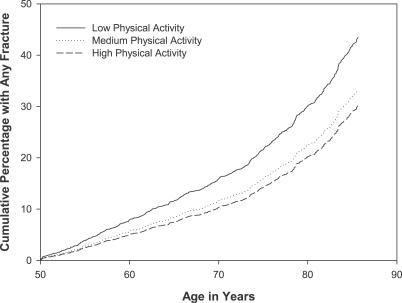
Survival Curves of Participants with Any Type of Fracture after Study Entry at 50 y of Age for the Time-Dependent Low, Medium and High Physical Categories Survival curves are adjusted by changes in physical activity during follow-up.

Unadjusted survival curves of hip fractures and all fractures with time-dependent exposure of physical activity are displayed in Figures 2 and 3, respectively. The absolute risk of hip fracture was highest among men with a sedentary lifestyle (Figure 2). Of these men, 20.5% had a hip fracture at the end of follow-up compared with 13.3% of the men with medium physical activity and 8.4% of the men with high physical activity. Regarding the number of men with any type of fracture after study entry (Figure 3), 43.6% of the men in the low physical category had suffered any type of fracture, compared with 33.3% in the medium physical activity category and 30.2% in the high physical activity category. There were small differences in the pattern of the survival curves if they were adjusted for the covariates (unpublished data).

Compared with men with a high physical activity level (Table 2), sedentary men had more than a doubled adjusted hip fracture risk (HR 2.56, 95% CI 1.55–4.24). Although lower than the adjusted hip fracture risk, the adjusted overall fracture risk of sedentary men was still increased (HR 1.57, 95% CI 1.20–2.07). It remained increased when the analysis was restricted to non-hip fracture patients (adjusted HR 1.48, 95% CI 1.10–2.00). Men with a medium activity level had a 60% higher hip fracture risk than those who reported regular sports activities (HR 1.61, 95% CI 1.10–2.36). The same comparison did not reveal a significant disparity in the overall fracture risk between these groups, however.

**Table 2:**
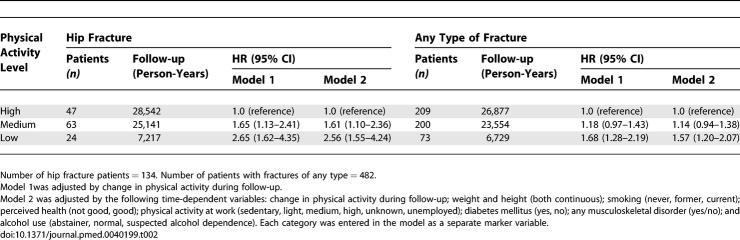
HRs with 95% CIs of Hip Fractures and any Type of Fracture Associated with Time-Dependent Physical Activity Level in Leisure Time

According to the estimation of the population-attributable risk, approximately one third of all hip fractures could have been prevented if all men had had a high physical activity level (crude estimate 35%, 95% CI 16%–51%; adjusted estimate 34%, 95% CI 14%–50%).

**Figure 4:**
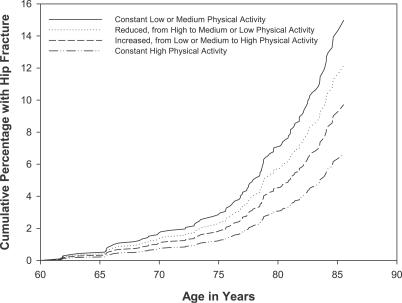
Survival Curves of Hip Fractures after Age 60 y by Change in Physical Activity between 50 and 60 y of Age Survival curves are adjusted by reported changes in physical activity at age 70, 77, and 82 y.

The results by change in physical activity between the first and the second investigation at age 60 y were analyzed, and the proportion of men with hip fractures within each category of changed physical activity are presented as survival curves in Figure 4. Approximately 38% of the men reported unchanged low or medium physical activity, 24% reported decreased activity from high to low or medium, 11% reported increased activity from low or medium to high, and 27% had a high physical activity at both these investigations. In the analysis, we also adjusted the estimates for later changes in activity. The lowest proportion of men with hip fractures was found among men who maintained a high physical activity, and we observed the highest proportion of men with hip fractures among men with a sustained low and medium physical activity. Amongst those men who had a changed activity level, intermediate proportions of hip fractures were found.

Compared with men who had a continuous high physical activity (Table 3), those who had a constant low or medium physical activity had an adjusted HR for hip fracture of 2.31 (95 % CI 1.39–3.85), whereas men who decreased their activity level from high to medium or from high to low had an adjusted HR of 1.94 (95 % CI 1.08–3.48). The men who reported an increase in activity from low and medium to a high level had an adjusted HR of 1.50 (95 % CI 0.78–2.86).

**Table 3:**
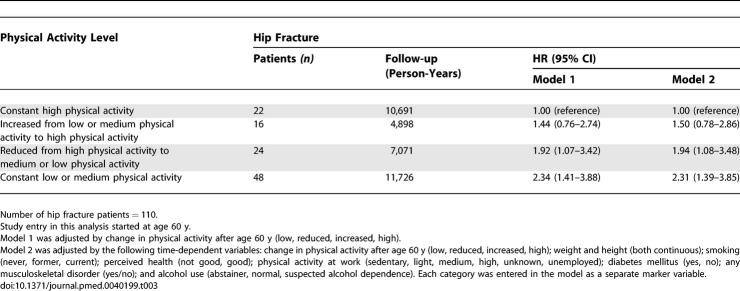
HRs with 95% CIs of Hip Fractures associated with a Change in Physical Activity Level in Leisure Time from 50 to 60 y of Age

## Discussion

Even if it is intuitively appealing that physical activity should prevent osteoporotic fractures, the scientific evidence for this opinion is unexpectedly weak for men [9–12]. Our results extend previous research by having the longest follow-up, the largest number of fractures, five repeated measures of physical activity, time-dependent analysis of the data, and validation of the physical activity categories by physiological measures.

Our data indicate that avoiding a low level of physical activity substantially reduces the risk of all fractures, particularly hip fractures—the most devastating of osteoporotic fractures—in men. Even changes in physical activity during the follow-up affected hip fracture risk. As expected, those who maintained a high physical activity level had the lowest risk of hip fracture, but there was also a tendency towards a lower risk of fracture for those who increased their level of activity compared with those who reduced their level of activity, or compared with those who reported constant low activity. This observation has previously been made in women [8,16]. There are several possible mechanisms, related to muscle performance and balance as well as to bone architecture and strength, whereby physical activity can reduce the risk of fractures [28,29].

Our population-based study had the advantage of monitoring men of comparable age from a time before they reached the ages most affected by a high incidence of fractures. The fractures and other diseases were identified from registers by use of the unique individual personal registration number given to all Swedish citizens. The repeated assessments throughout the follow-up period enabled us to take health and lifestyle changes into account in the analysis. We believe that this analytical approach is an important improvement on earlier studies since our risk estimates were attenuated if only baseline data were used (unpublished data).

Participants at the later investigations were probably more physically active than non-participants. Nevertheless, the consequences of this misclassification of non-participants would only lead to an underestimation of the actual effect of physical activity, as would any incorrect self-reporting of physical activity. A low proportion of participants reported hard physical training, and these participants were not considered separately, but there did not seem to be a further reduction in fracture risk: adjusted HR of hip fracture 0.94 (95% CI 0.34–2.63) and of all fractures 1.16 (95% CI 0.74–1.83) for strenuous activity versus recreational sports. Physical activity at work was not associated with fracture risk (unpublished data) and did not have an impact on our leisure physical activity estimates.

In conclusion, we found a graded reduction of the fracture risk with increasing physical activity in men. We suggest that older men should increase their leisure physical activity to reduce their risk of osteoporotic fractures.

## Abbreviations

CI: confidence interval
HR: hazard ratio, ULSAM, Uppsala Longitudinal Study of Adult Men
VO_2_max: maximal oxygen uptake
